# Intestinal-type adenocarcinoma presenting as a female periurethral mass

**DOI:** 10.1186/s12894-022-01077-6

**Published:** 2022-08-01

**Authors:** Ting Zeng, Qiang Liu, Shuxia Zhu, Ruihua Xu

**Affiliations:** 1 West China Hospital, Sichuan University, No. 37 Guo Xue Xiang, Sichuan 610041 Chengdu, People’s Republic of China; 2 Chengdu Second People’s Hospital, No. 10 Qingyun Nan, Sichuan 610041 Chengdu, People’s Republic of China

**Keywords:** Periurethral mass, Intestinal-type adenocarcinoma, Case report

## Abstract

**Background:**

The periurethral mass in the female is a rare clinical entity and most of the lesions are benign. We present an unusual case of a periurethral mass found to be intestinal-type adenocarcinoma which has not been previously reported in the literature.

**Case presentation:**

A 58-year-old woman was referred to our hospital with acute urinary retention. She complained of frequency, urgency and progressive obstructive urinary symptoms for the last 3 months. A pelvic magnetic resonance imaging scan showed a soft tissue mass of 5 × 4 cm surrounding the entire urethra. A needle biopsy was done and revealed adenocarcinoma with intestinal-type features. The tumor was removed by a simultaneous laparoscopic abdominal and transperineal approach. The pathological results showed a positive surgical margin and urethra and vagina wall invasion. The neoplastic cells were positive for CK20, CDX-2, CerbB-2, MSH2, MSH6, MLH1, PMS2 and P53. The patient received adjuvant systemic chemotherapy comprising S-1 and oxaliplatin. Follow-up with pelvic MRI 6 months after surgery showed no signs of local recurrence.

**Conclusions:**

We have reported the first case of the primary periurethral adenocarcinoma of intestinal type. There are currently no standardized protocols for the diagnosis, clinical course, and treatment of this rare tumor. This case study can aid decision-making regarding the diagnosis and treatment of this tumor.

## Background

The periurethral mass in the female is a rare clinical entity. However, the differential diagnosis of the mass is extensive as it comprise a wide spectrum of pathologies. The most frequently encountered periurethral masses are Urethral diverticulum, leiomyomas, vaginal cyst, Skene duct cysts, leiomyoma and fibromyoma [1, 2].

Intestinal-type adenocarcinoma is a high-grade malignancy which is most frequently localized in the ethmoid sinus (40%), the nasal cavity (25%) and the maxillary antrum (20%) [3, 4]. The primary intestinal-type adenocarcinoma arising in the genitourinary tract is extremely rare. We herein present an unusual case of intestinal-type adenocarcinoma presenting as a female periurethral mass.

## Case presentation

A 58-year-old woman was referred to our hospital in September 2021 with acute urinary retention. She complained of frequency, urgency and progressive obstructive urinary symptoms for the last 3 months. She denied pain, haematuria or bleeding. Her surgical history revealed a hysterectomy due to hysteromyoma 8 years prior. Her past medical and family histories were unremarkable.

**Fig. 1:**
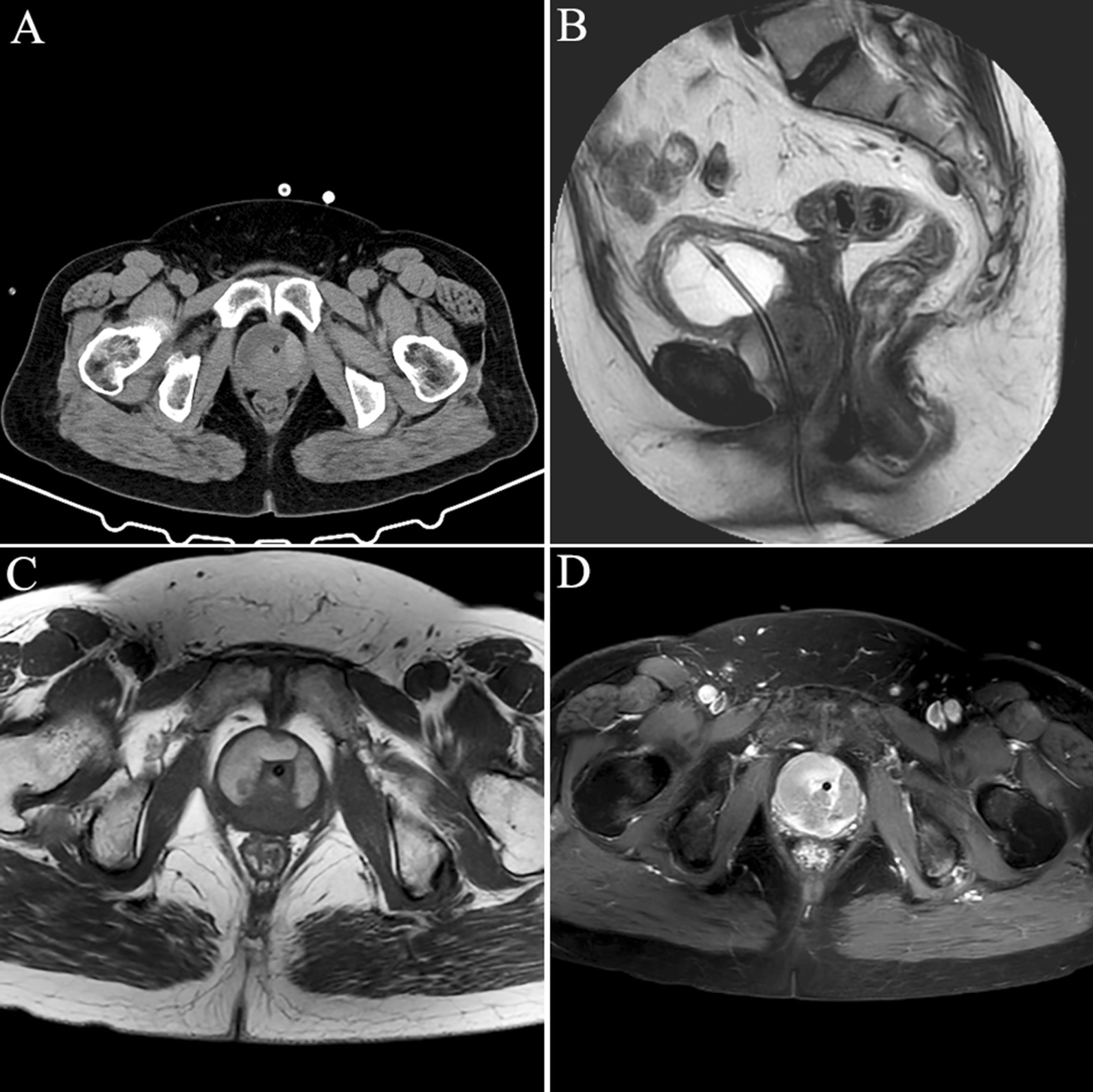
The CT and MRI features of the periurethral mass. **a** Pelvic CT scan; **b** sagittal T2-weighted MRI image; **c** axial T1-weighted MRI image; **d** axial T1 image with contrast

**Fig. 2:**
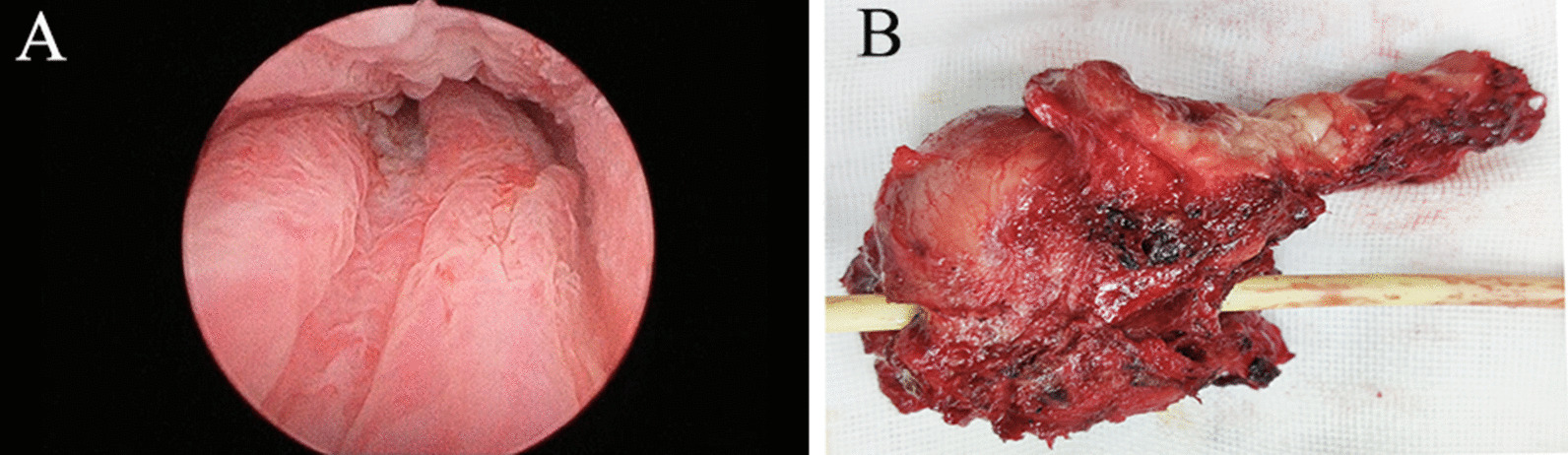
Cystourethroscopy result and gross appearance of periurethral mass. **a** Cystourethroscopy revealed normal urethral appearance; **b** gross examination of the resected specimen revealed a solid mass (5 × 4 × 3 cm) with irregular margins

The ultrasound in emergency department showed urinary retention, but no other abnormalities were found. To find the possible cause of urinary retention, we performed a pelvic Computerized Tomography (CT) scan and a periurethral mass was found (Fig. 1a). On physical examination, an immobile hard mass was palpated on the ventral wall of the vagina, suggestive of a solid tumor. The inguinal lymph nodes were not enlarged on palpation. A pelvic magnetic resonance imaging (MRI) scan was then performed which showed a soft tissue mass of 5 × 4 cm surrounding the entire urethra (Fig. 1). Cystourethroscopy was performed which revealed no urethral or intravesical invasion (Fig. 2a). A needle biopsy was done and revealed adenocarcinoma with intestinal-type features (CDX-2+ CK20+, CEA+). To rule out the primary colorectal adenocarcinoma, the patient subsequently underwent abdomen CT scan and gastrointestinal endoscopy which showed no evidence of gastrointestinal primary tumors and disseminated disease.

**Fig. 3:**
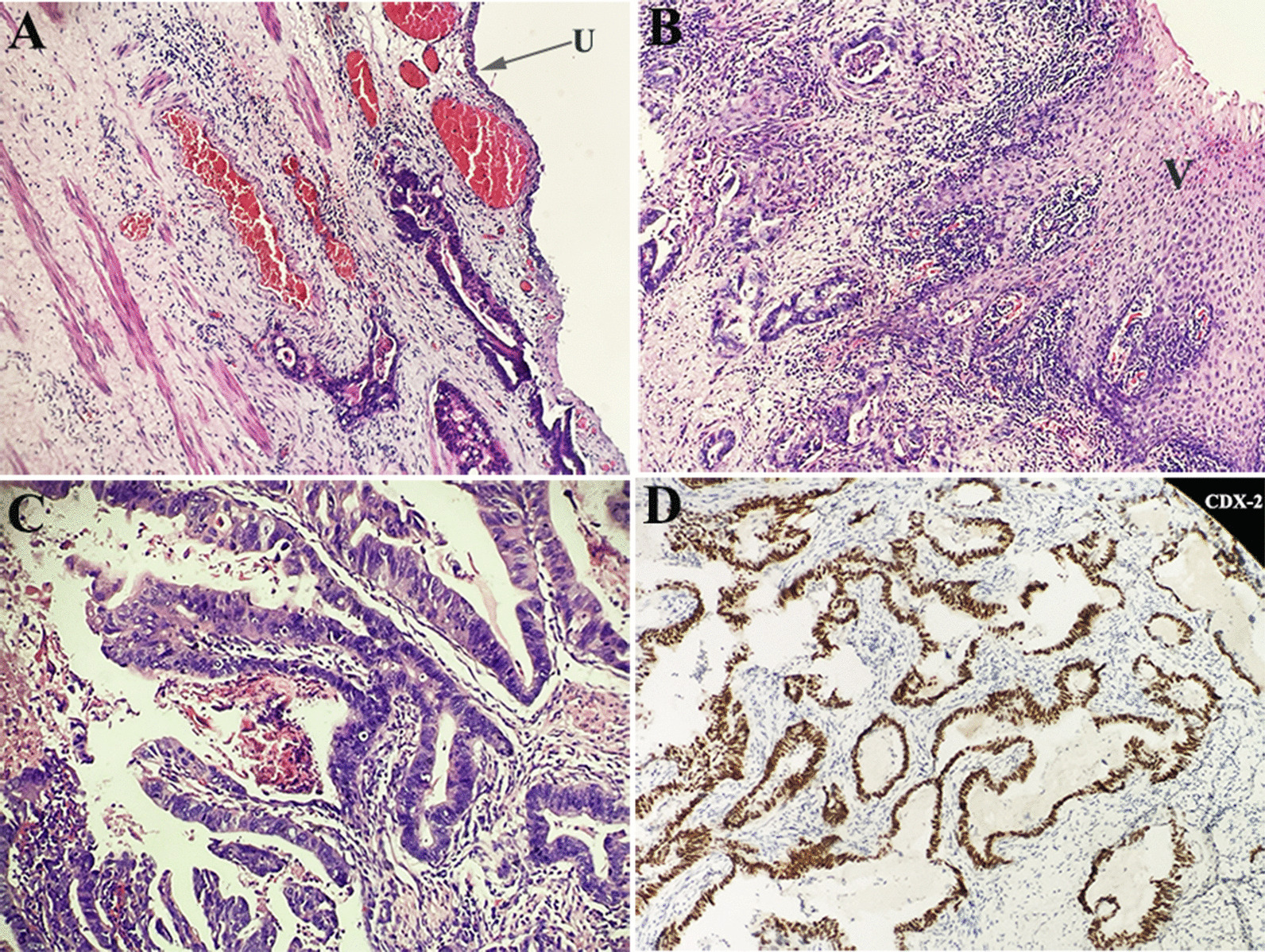
Histopathology. **a** Hematoxilin eosin staining, glandular structures with intracytoplasmatic mucin; **b** the tumor invaded the vaginal wall; **c** the tumor cells arranged in glands suggestive of colonic type of intestinal-type adenocarcinoma; **d** Immunohistochemistry, positive for CDX-2. U, urethral; V, vagina

Considering the high malignancy of this tumor, radical cystourethrectomy was suggested. But the patient refused the plan and insisted on bladder preservation. The tumor was removed by a simultaneous laparoscopic abdominal and transperineal approach. The mass was firmly adherent to its surrounding tissue and part of the anterior vagina wall was resected. Gross examination of the resected specimen revealed a solid mass (5 × 4 × 3 cm) with irregular margins (Fig. 2b). The pathological results showed a positive surgical margin and urethra and vagina wall invasion (Fig. 3). Immunohistochemically, neoplastic cells were positive for CK20, CDX-2, CerbB-2, MSH2, MSH6, MLH1, PMS2 and P53. The patient’s postoperative course was uneventful. The patient received adjuvant systemic chemotherapy comprising S-1 and oxaliplatin (SOX, oxaliplatin 200 mg [day 1] and S-160 mg bid of a 3-week cycle). The chemotherapy went smoothly and the patient showed no obvious discomfort. Follow-up with pelvic MRI 6 months after surgery showed no signs of local recurrence.

## Discussion and conclusions

Periurethral mass in women is an uncommon diagnosis, which is a challenge to the medical professionals due to the nonspecific clinical presentation and a broad differential diagnosis. Urethral diverticulum is the most common pathology (84%), whereas vaginal cyst (6%) and leiomyoma (7%) comprise the majority of the remaining pathologies [1]. Most of the periurethral lesions are benign and malignant lesions are extremely rare. Although the risk of malignancy is small, differentiating between benign and malignant lesions is important for management and surveillance. Imaging techniques including MRI and CT can provide useful information to aid diagnosis [5]. when a preliminary diagnosis cannot be made based on physical examination and radiologic studies, biopsy should be performed.

Intestinal-type adenocarcinomas are aggressive malignancies which are most frequently localized in in the sinonasal tract. To our knowledge, this is the first reported case of intestinal-type adenocarcinoma arising in the periurethral region. In present case, immunohistochemical stains showed positive reaction for CK20 and CDX2, supporting the diagnosis of intestinal-type adenocarcinoma. As the tumor mimic the appearances of carcinomas or adenomas of intestinal origin, extensive research including gastrointestinal tract fiberscope, cystoscopy and CT scan should be performed to rule out the primary colorectal adenocarcinoma.

Due to the rarity of this tumor, there is no data in literature regarding strategy for their optimal treatment. As Intestinal-type adenocarcinomas behave as high-grade malignancies [4], radical cystourethrectomy with a wide resection of the paraurethral tissue and anterior vaginal wall, may offer superior local control. In this case, the patient refused radical cystourethrectomy and received surgical resection of the periurethral mass with entire urethra and part of the anterior vagina wall. Since the distal end of the mass is firmly adjacent to the vaginal wall, transperineal approach may be necessary for the complete excision of the mass. For patients with locally advanced disease, a multimodal therapeutic approach is required. In this case, the palliative chemotherapy was administered to our patient because of positive surgical margin. The inclusion of systemic therapy may offer improvement of local control and reduction of distant metastasis.

In conclusion, we have reported the first case of the primary periurethral adenocarcinoma of intestinal type. There are currently no standardized protocols for the diagnosis, clinical course, and treatment of this rare tumor. This case study can aid decision-making regarding the diagnosis and treatment of this tumor.

## Data Availability

Data sharing is not applicable to this article as no datasets were generated or analysed during the current study.

## Abbreviations

CT: Computed tomography
MRI: Magnetic resonance imaging
